# Low Admission Immunoglobulin G Levels Predict Poor Outcome in Patients with Mild-to-Critical COVID-19: A Prospective, Single-Center Study

**DOI:** 10.1007/s44197-021-00002-8

**Published:** 2021-08-10

**Authors:** Charikleia S. Vrettou, Alice G. Vassiliou, Ioannis Kakkas, Edison Jahaj, Stamatios Tsipilis, Nikolaos Athanasiou, Alexandros Zacharis, Chrysi Keskinidou, Aikaterini Papageorgiou, Stylianos E. Orfanos, Anastasia Kotanidou, Ioanna Dimopoulou

**Affiliations:** 1 First Department of Critical Care Medicine and Pulmonary Services, School of Medicine, National and Kapodistrian University of Athens, “Evangelismos” Hospital, 45-47 Ipsilantou St, 10676 Athens, Greece; 2 Department of Immunology and Histocompatibility, “Evangelismos” Hospital, 45-47 Ipsilantou St, 10676 Athens, Greece

**Keywords:** Immunoglobulin G, COVID-19, Mortality, Intensive care unit

## Abstract

**Introduction:**

Immunoglobulins (Igs) comprise a critical part of the immune response. Little information exists on Ig serum levels in COVID-19 patients. We, therefore, investigated whether hospital admission Igs in patients with mild-to-critical disease are associated with clinical outcome.

**Materials and Methods:**

This prospective, observational, single-center, cross-sectional study included 126 consecutive non-critically ill and critically ill and COVID-19 patients, in whom IgG, IgM, and IgA were measured on hospital admission.

**Results:**

The cohort was divided in survivors and non-survivors, based on in-hospital mortality. Median IgG levels of survivors were significantly higher than non-survivors (*p* < 0.01). The cohort was subsequently divided in IgG deficient (< 690 mg/dl) and sufficient (≥ 690 mg/dl) patients. IgG-deficient patients had a higher mortality rate (*p* < 0.01). The multivariate logistic regression model showed that subnormal IgG was significantly associated with increased mortality risk (*p* < 0.01).

**Conclusion:**

In our COVID-19 cohort, admission subnormal IgG levels might be independently associated with reduced survival.

## Introduction

Coronavirus disease 2019 (COVID-19) is caused by the severe acute respiratory coronavirus 2 (SARS-CoV-2). While the majority of infected individuals experience only mild symptoms or are even asymptomatic, about 10 to 20% of patients rapidly progress to acute respiratory distress syndrome (ARDS), and multiple organ dysfunction, requiring treatment in the intensive care unit (ICU). A few possible treatment choices exist at the moment, yet the requirement to search for better therapeutic options remains persistent [1].

In general, the severity of an infection depends on the virulence of the pathogen, and the immunological response of the host. Although an active immune response is essential to eliminate pathogens, uncontrolled host immune reactions account for damage of healthy cells and tissues, determining subsequent outcome. So far, the pathophysiology and the unusually high pathogenicity of COVID-19 remain incompletely understood. Cytokine release syndrome, and lymphopenia are features of patients with severe COVID-19, indicating an increased systemic inflammatory response [2]. Immunoglobulins (Igs), produced by plasma cells, act as a critical part of the overall immune response, and are particularly effective in the identification, neutralization, opsonization, and direct lysis of pathogens. They additionally possess anti-inflammatory and immunomodulatory properties [3]. However, there is little information on changes in serum levels of Igs in patients diagnosed with COVID-19 [4, 5], in particular in those treated in the ICU [6]. Moreover, the impact of endogenous Igs on the prognosis of patients with COVID-19 has not been sufficiently explored and inconsistent results have been reported [7, 8].

Given this background, the aim of this prospective study was to investigate whether Igs (IgG, IgM, and IgA) measured on admission in the hospital in patients with mild-to-critical COVID-19 are a risk factor for mortality.

## Materials and Methods

This prospective, observational, single-center, cross-sectional study included consecutive adult non-critically and critically ill COVID-19 patients, admitted to the “Evangelismos” Hospital from September 18^th^ 2020 to December 14^th^ 2020. SARS-CoV-2 infection was diagnosed by real-time reverse transcription PCR (RT-PCR) in nasopharyngeal swabs. Patients with known, pre-existing immunodeficiency states were excluded. The study was approved by the Hospital’s Research Ethics Committee (360/17-9-2020), and all procedures carried out on patients were in compliance with the Helsinki Declaration. Informed written consent was obtained from all patients or patients’ next-of-kin.

Following study enrollment, demographics, comorbidities, symptoms, and laboratory data were recorded. Additionally, in critically ill patients, Acute Physiology and Chronic Health Evaluation (APACHE II) and Sequential Organ Failure Assessment (SOFA) scores were calculated on admission in the ICU. Outcome was defined as in-hospital mortality.

### Immunoglobulin Measurements

Four milliliters (4 ml) of venous blood were collected within the first 24–48 h following hospital admission. Serum was drawn in BD Vacutainer® Plus Plastic Serum Tubes. Serum was collected, portioned into 0.5 ml aliquots, and stored at − 80 °C until analyzed. Nephelometry (Beckman Coulter, USA) was used to detect the levels of IgG, IgM and IgA. Normal ranges, according to our Immunology and Histocompatibility Department, are as follows: IgG: 690–1680 mg/dl; IgM: 40–235 mg/dl; IgA: 72–400 mg/dl.

### Statistical Analysis

Data are presented as individual values, mean ± standard deviation (SD) for normally distributed variables, and median with interquartile range (IQR) for variables with skewed distribution. Two groups comparisons were performed by the *t* test or the non-parametric Mann–Whitney test for skewed data. Associations between qualitative variables were examined by the chi-squared test. Correlations were performed by Spearman’s correlation coefficient. A univariate logistic regression model was fitted to examine the relationship of IgG levels with in-hospital mortality. A multivariate logistic regression model was subsequently performed to adjust for statistically significant variables by the two-group comparisons (namely age, white blood cell count, percentage of lymphocytes, D-dimers, lactate dehydrogenase and fibrinogen, continuous variables, and sex, categorical). A receiver operating characteristic (ROC) curve was plotted using in-hospital mortality as the classification variable and IgG levels on hospital admission, and their linear combination with age, as prognostic variables. The analyses were performed with IBM SPSS statistical package, version 22.0 (IBM Software Group, New York, USA), and GraphPad Prism, version 8.0 (GraphPad Software, San Diego, USA). All the *p *values were calculated after two-sided tests; *p *values < 0.05 were considered significant.

## Results

**Fig. 1:**
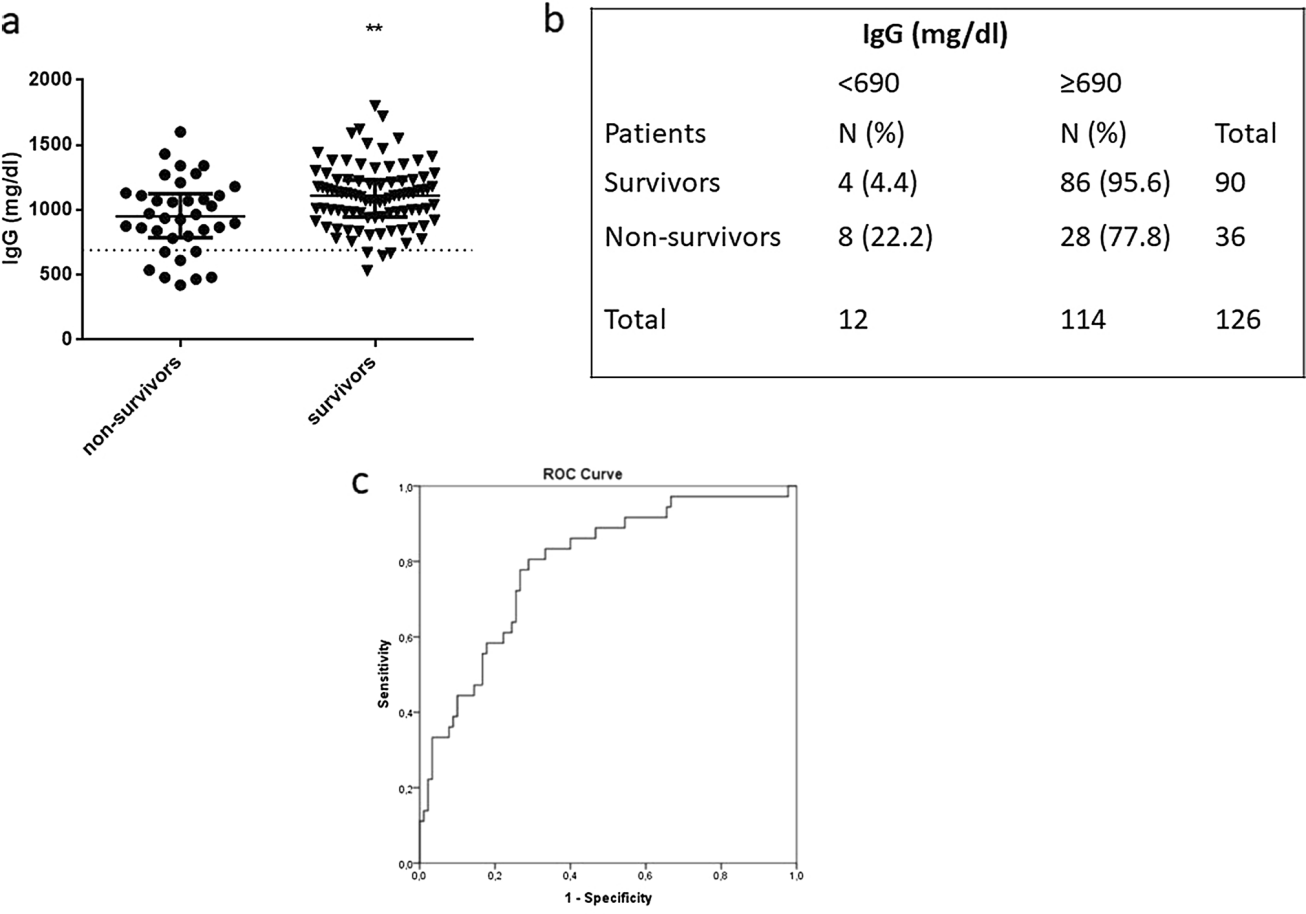
IgG levels and mortality. **a**–**b** IgG levels were measured in COVID-19 hospitalized patients on hospital admission (*N* = 126). Patients were subsequently categorized as survivors (*N* = 90) and non-survivors (*N* = 36). IgG levels on admission were compared between the two groups. A difference was observed in IgG levels of the two groups (**a**: IgG continuous, *p* = 0.008; **b**: IgG categorical, *p* = 0.005). Two-group comparisons were performed using the non-parametric Mann–Whitney test for skewed data for continuous IgG variable and chi-squared test for categorical IgG variable with two categories (cut-off = 690 mg/dl). Data are presented as scatter plots. Line in the middle, median value; lower and upper lines, 25th to 75th centiles; horizontal line, cut-off value for IgG deficiency (690 mg/dl) (**a**). Data are expressed as number of patients (*N*) and percentages of totals (%) (**b**). **c** Receiver operating characteristic (ROC) curve analysis. A ROC curve was generated to determine the prognostic accuracy of IgG combined with age to predict in-hospital mortality. The corresponding area under the curve (AUC) and 95% confidence intervals (CI) were estimated as follows: 0.792 (0.705–0.878, *p* < 0.0001)

The cohort consisted of 126 consecutive patients (82 males and 44 females) with a mean age of 62 ± 15 years. Of these, 74 patients were hospitalized in the ward, and 52 in the ICU. The vast majority (67%) had comorbidities. In critically ill patients, median values for APACHE II and SOFA were 15 and 6, respectively. The patients presented with symptoms 6 days prior to hospital admission. Of the total of 126 patients, 36 patients died, yielding a mortality rate of 28.6%. Ten patients (13.5%) died in the ward, while 26 patients (50%) in the ICU. The cohort was divided in survivors and non-survivors, based on in-hospital mortality. Demographics and laboratory data of the two patient groups are summarized in Table 1. As seen, variables that differed between the two groups were age, sex, white blood cell count, percentage of lymphocytes, fibrinogen, D-dimers and lactate dehydrogenase (LDH). Most importantly, median IgG levels of the survivors were significantly higher than those of the non-survivors (1110 mg/dl vs. 951 mg/dl; *p* = 0.008; Fig. 1a), however, IgA and IgM were similar in the two groups. Spearman’s correlations indicated that IgG levels correlated with IgA levels (*r*_s_ = 0.38, *p* < 0.0001), while they did not correlate with IgM levels (*r*_s_ = 0.08, *p* > 0.05). IgM and IgA levels also did not correlate (*r*_s_ = − 0.008, *p* > 0.05). As expected, IgG correlated with total proteins (*r*_s_ = 0.34, *p* < 0.001) and globulins (*r*_s_ = 0.46, *p* < 0.0001).

**Table 1:** Demographics, laboratory data, and immunoglobulins in survivors and non-survivors on hospital admission

Characteristics	Survivors	Non-survivors	*p* value	Reference values
Number of patients, *N* (%)	90 (71.4)	36 (28.6)		
Age (years), (mean ± SD)	58 ± 13	71 ± 15	< 0.0001*	
Sex, *N* (%)			0.02*	
Male	53 (58.9)	29 (80.6)		
Female	37 (41.1)	7 (19.4)		
Comorbidities, *N* (%)	56 (62.2)	28 (77.8)	0.1	
Laboratory data				
White blood cell count (per μl), (median, IQR)	6590 (4760–103,210)	8760 (4540–16,100)	0.02*	4–10.5 × 10^3^
Neutrophils (%), (median, IQR)	75.0 (60.6–84.0)	88.0 (72.4–91.7)	0.08	40–70
Lymphocytes (%), (median, IQR)	21.0 (9.3–31.0)	7.1 (4.5–14.2)	0.02*	25–45
Platelets (per μl), (median, IQR)	233,000 (174,000–289,000)	203,000 (165,000–258,000)	0.3	140–450 × 10^3^
Fibrinogen (mg/dl), (mean ± SD)	530 ± 150	628 ± 231	0.008*	200–400
D-dimers (ng/ml), (median, IQR)	0.69 (0.43–1.04)	1.96 (0.76–3.06)	0.004*	< 0.5
LDH (U/L), (median, IQR)	289 (218–400)	376 (285–500)	0.03*	< 225
PCT (ng/ml), (median, IQR)	0.11 (0.07–0.28)	0.30 (0.12–1.67)	0.9	< 0.05
Ferritin (ng/ml), (median, IQR)	320 (145–628)	471 (268–1513)	0.2	12–263
CRP (mg/dl), (median, IQR)	6.4 (2.0–10.2)	13.1 (6.3–23.3)	0.3	< 0.5
Globulin (g/dl), (median, IQR)	2.6 (2.4–3.0)	2.6 (2.4–3.0)	0.8	2.3–3.5
Immunoglobulins (Igs)				
IgG (mg/dl), (median, IQR)	1110 (946–1230)	951 (787–1125)	0.008*	690–1680
IgM (mg/dl), (median, IQR)	96 (67–147)	81 (60–125)	0.2	40–235
IgA (mg/dl), (median, IQR)	228 (172–290)	268 (172–350)	0.1	72–400
Length of hospital stay (days), (median, IQR)	10 (7–12)	20 (11–27)	< 0.0001*	

*p value < 0.05. Data are expressed as number of patients (*N*) and percentages of total related variable (%), mean ± SD for normally distributed variables, or median (IQR) for skewed data. Patients were divided in two groups depending on in-hospital mortality. For differences between the two groups, either the Student’s *t* test for normally distributed data or the Mann–Whitney test for skewed data was used. Associations between qualitative variables were examined by the chi-squared test. Laboratory data were measured once (within 24–48 h from admission). In the total of 126 patients, 12 subjects had IgG values below the normal reference range of our laboratory (690 mg/dl), while 2 had values above the maximum value (1680 mg/dl). For IgM, 4 patients had values below the normal reference range (40 mg/dl), while 1 had values above the maximum value (235 mg/dl). For IgA, five patients had values below the normal reference range (72 mg/dl), while 16 had values above the maximum value (400 mg/dl). CRP, C-reactive protein; Ig, immunoglobulin; LDH, lactate dehydrogenase; PCT, procalcitonin

We subsequently divided our cohort in IgG deficient (< 690 mg/dl) (*N* = 12) and sufficient (≥ 690 mg/dl) (*N* = 114) patients. IgG deficient patients had a higher mortality rate (78% vs. 22%, *p* = 0.005). Hence, among hospitalized patients, both continuous and categorical IgG levels on hospital admission differed between survivors and non-survivors. Figure 1 depicts the difference observed in IgG levels on hospital admission (continuous; *p* = 0.008; Fig. 1a and categorical; *p* = 0.005; Fig. 1b).

To further explore the associations between admission IgG levels and mortality risk, we performed logistic regression analysis. In univariate analysis, low IgG levels were associated with increased mortality risk. The odds ratio was 0.996 (95% CI = 0.993–0.999; *p* = 0.013). The multivariate model controlled for potential confounding factors, including, age, white blood cell count, percentage of lymphocytes, fibrinogen, LDH, and D-dimers (continuous variables), and sex (categorical). Multivariate model analysis raises the possibility that low IgG levels might be an independent predictor of poor outcome (adjusted OR = 0.996, CI = 0.994–0.999; *p* = 0.007), in the presence of age, sex, and white blood cell count.

A ROC curve was generated to determine the prognostic accuracy of IgG in predicting in-hospital mortality; the area under the curve (AUC) of IgG levels was 0.650 (95% CI = 0.540–0.761, *p* = 0.009). Additionally, we investigated the predictive value of IgG levels combined with age. The AUC of the combined ROC was 0.792 (0.705–0.878, *p* < 0.0001; Fig. 1c).

## Discussion

To our knowledge, this is the largest prospective study presenting admission serum levels of Igs (IgG, IgM, and IgA) in adult patients with COVID-19 with the entire range of disease severity. We observed that non-survivors had lower IgG levels than survivors, and that IgG deficient patients had higher mortality rates compared to non-deficient patients. Low IgG was an independent predictor for poor outcome. In contrast, IgM and IgA were not linked to survival. Taken together, these suggest that low admission IgG might predict subsequent poor survival in patients with mild-to-critical COVID-19.

Since the first reports of COVID-19, a plethora of prognostic factors have been reported in the still growing literature, including demographics, comorbidities, along with hematologic, cardiac, renal, inflammatory, and coagulation biomarkers [9]. Indeed, in our study, male sex, age, neutrophilia, lymphopenia, elevated D-dimers, fibrinogen, and LDH, were associated with an increased risk for death. A limited number of studies have focused on serum Ig levels with respect to COVID-19 severity and clinical outcome. To note, contradictory results have been presented. This might be related to the fact that most information is derived from retrospective studies and variable cut-off levels have been used for hypo-Ig definitions. Qin *et al*. recruited 452 patients with severe or mild COVID-19. IgM was significantly lower in severe cases, but there were no differences in IgA or IgG between the two groups [5]. On the other hand, a subsequent study showed different results [4]. Data on 276 patients with mild, moderate or severe disease were analyzed. A group of healthy volunteers was also included for comparison. At the time of admission, COVID-19 patients had serum concentrations of IgG, IgA or IgM isotypes comparable to those in healthy volunteers. However, when patients with different degrees of severity were assessed separately, it was observed that, in the severe cases, the levels of IgA and IgM were similar, but the IgG concentrations were lower compared to healthy volunteers. A few studies attempted to link Ig levels with survival in COVID-19. A study on 125 patients showed that the concentrations of IgG, IgA, and IgE were increased in non-survivors compared to survivors, whereas IgM levels did not differ [8]. A subsequent study enrolled 236 patients and confirmed that non-survivors had higher IgA and IgE than survivors, however, IgG was similar in the two groups [7].

Our study included COVID-19 patients with the whole range of disease severity, *i.e.*, cases hospitalized in the ward with mild to severe infection, along with critically ill patients treated in the ICU. Moreover, our study *per design* was prospective and we measured consistently Ig levels at a predetermined time point, early (within 48 h) following hospital admission. This ensured that administered drugs and co-infections from other pathogens, in particular in ICU patients, did not interfere with Ig levels. We found that IgG concentrations of the non-survivors were significantly lower than those of the survivors. We subsequently divided our cohort in IgG deficient and sufficient patients. IgG deficiency was noted in about 10% of the cohort and these patients had a higher mortality rate compared to their IgG non-deficient counterparts. Moreover, low initial IgG was independently associated with an increased mortality risk. Contrasting previous investigations [7, 8], we did not observe differences in IgA and IgM between survivors and non-survivors. Our results are in agreement with a recent prospective study with respect to low IgG levels and high mortality rates, which however, was carried out in a smaller cohort of ICU patients (*N* = 62) [6]. In that study IgG deficiency was identified in 21% of patients and was associated with more severe disease, and higher mortality rates. It remains unclear whether low IgG levels observed in our study is a hallmark of COVID-19 *per se*, since low IgG has been described in other infectious states, such as community-acquired pneumonia and ICU septic patients [10, 11]. Mechanisms underlying low IgG remain obscure, and may include diminished IgG production, vascular leakage, or utilization of Igs by the complement system [11]. Alternatively, the possibility that some patients had a pre-existing, undiagnosed immunodeficiency cannot be excluded.

Innate and acquired immune responses vary according to the severity of COVID-19 infection, and have been linked to clinical outcome. More specifically, the severe form of COVID-19 has been attributed to a dysfunctional innate immune response, including deficient type I interferon response, coupled with an exaggerated adaptive immunity. Severe COVID-19 patients exhibit a significantly reduced number of natural killer cells, while complement activation regulates a systemic pro-inflammatory response to SARS-CoV2 infection. In adaptive immunity, differences in T-cell and B-cell responses have been identified in patients with severe disease compared to mild cases [12–15]. Regardless of mechanisms, IgG constitutes an important component of humoral immunity, and its presence is essential to fight pathogens. IgG is the most abundant Ig and exerts its multiple beneficial immune functions through its Fab and Fc domains [3].

Treatment for COVID-19 can mainly be divided into two types depending on the targets: SARS-CoV-2, and systemic inflammation induced by the virus. Intravenous immunoglobulin (IVIG) contains human Igs, mainly IgG, that are pooled from plasma of healthy donors. The compound provides passive immunity and modulates immune functions. Currently, IVIG is widely used in life-threatening infections in patients with primary and secondary immune deficiencies, and autoimmune/inflammatory disorders [16]. The possible effects of IVIG to fight viral infections, including COVID-19, has been recently reviewed and highlighted that there are many limitations for evaluating its efficacy [17]. It remains currently unclear whether pre-evaluation of endogenous Ig levels is a valid biomarker to target COVID-19 patients suitable for adjunct treatment with IVIG.

The limitations of the present study should be acknowledged. Firstly, the number of patients was relatively small, but larger compared to other prospective studies [6]. The OR (95% CI) of IgG levels from the multivariate model analysis were close to 1, however statistically significant. This may be related to the small sample size. We did not calculate the sample size prior to the study. The power of the study was calculated *post hoc* and the observed power was 84.5%. The study was single-centered, and the generalization of our results remains to be demonstrated. Finally, single Ig measurements (on admission) were performed. Serial Ig analysis might better identify IgG deficient patients, since the nadir in its levels may have occurred later during the course of the disease.

## Conclusion

In our cohort, subnormal endogenous IgG levels in mild-to-critical adult patients with COVID-19 might be independently associated with a reduced survival. Whether measurement of IgG could be used as a stratification marker for IVIG therapy to prevent disease progression and improve patients’ prognosis needs to be investigated.

## Data Availability

The data that support the findings of this study are available from the corresponding author upon reasonable request.

## References

[1] Mehta OP, Bhandari P, Raut A, Kacimi SEO, Huy NT (2020). Coronavirus disease (COVID-19): comprehensive review of clinical presentation. Front Public Health.

[2] Celardo I, Pace L, Cifaldi L, Gaudio C, Barnaba V (2020). The immune system view of the coronavirus SARS-CoV-2. Biol Direct.

[3] Schroeder HW, Cavacini L (2010). Structure and function of immunoglobulins. J Allergy Clin Immunol.

[4] Marcos-Jiménez A, Sánchez-Alonso S, Alcaraz-Serna A (2020). Deregulated cellular circuits driving immunoglobulins and complement consumption associate with the severity of COVID-19 patients. Eur J Immunol.

[5] Qin C, Zhou L, Hu Z (2020). Dysregulation of immune response in patients with coronavirus 2019 (COVID-19) in Wuhan. China Clin Infect Dis.

[6] Husain-Syed F, Vadász I, Wilhelm J (2020). Immunoglobulin deficiency as an indicator of disease severity in patients with COVID-19. Am J Physiol Lung Cell Mol Physiol.

[7] Fang S, Wang H, Lu L, Jia Y, Xia Z (2020). Decreased complement C3 levels are associated with poor prognosis in patients with COVID-19: a retrospective cohort study. Int Immunopharmacol.

[8] Zhao Y, Nie HX, Hu K (2020). Abnormal immunity of non-survivors with COVID-19: predictors for mortality. Infect Dis Poverty.

[9] Kermali M, Khalsa RK, Pillai K, Ismail Z, Harky A (2020). The role of biomarkers in diagnosis of COVID-19—a systematic review. Life Sci.

[10] de la Torre MC, Torán P, Serra-Prat M (2016). Serum levels of immunoglobulins and severity of community-acquired pneumonia. BMJ Open Respir Res.

[11] Shankar-Hari M, Culshaw N, Post B (2015). Endogenous IgG hypogammaglobulinaemia in critically ill adults with sepsis: systematic review and meta-analysis. Intensive Care Med.

[12] Fletcher-Sandersjöö A, Bellander BM (2020). Is COVID-19 associated thrombosis caused by overactivation of the complement cascade? A literature review. Thromb Res.

[13] Hasan A, Al-Ozairi E, Al-Baqsumi Z, Ahmad R, Al-Mulla F (2021). Cellular and humoral immune responses in Covid-19 and immunotherapeutic approaches. ImmunoTargets Ther.

[14] Maecker HT (2021). Immune profiling of COVID-19: preliminary findings and implications for the pandemic. J Immunother Cancer.

[15] Mishra KP, Singh AK, Singh SB (2020). Hyperinflammation and Immune Response Generation in COVID-19. NeuroImmunoModulation.

[16] Perez EE, Orange JS, Bonilla F (2017). Update on the use of immunoglobulin in human disease: a review of evidence. J Allergy Clin Immunol.

[17] Moradimajd P, Samaee H, Sedigh-Maroufi S, Kourosh-Aami M, Mohsenzadagan M (2020). Administration of intravenous immunoglobulin in the treatment of COVID-19: a review of available evidence. J Med Virol.

